# Ketamine Administration in Prehospital Combat Injured Patients With Traumatic Brain Injury: A 10-Year Report of Survival

**DOI:** 10.7759/cureus.9248

**Published:** 2020-07-17

**Authors:** Allee C Torres, Vikhyat S Bebarta, Michael D April, Joseph K Maddry, Paco S Herson, Emma K Bebarta, Steven Schauer

**Affiliations:** 1 Emergency Medicine, University of Colorado School of Medicine, Aurora, USA; 2 Emergency Medicine, University of Colorado Anschutz Medical Campus, Aurora, USA; 3 Emergency Medicine, San Antonio Uniformed Services Health Education Consortium (SAUSHEC), Fort Sam Houston, USA; 4 Emergency Medicine, Brooke Army Medical Center, Fort Sam Houston, USA; 5 Military and Emergency Medicine, Uniformed Services University, Bethesda, USA; 6 Anesthesiology, University of Colorado School of Medicine, Aurora, USA; 7 Other, Cherry Creek High School, Greenwood Village, USA; 8 Office of the Senior Scientist, US Army Institute of Surgical Research, San Antonio, USA; 9 US Army Institute of Surgical Research, Joint Base Sam Houston, San Antonio, USA

**Keywords:** ketamine, head, trauma, military, prehospital, tccc

## Abstract

Background

The Tactical Combat Casualty Care (TCCC) guidelines recommend ketamine as the primary battlefield analgesic in the setting of moderate-to-severe pain and hemodynamic compromise. However, despite recent studies failing to support the association between ketamine and worse outcomes in head trauma, TCCC guidelines state that ketamine may worsen severe traumatic brain injury. We compared mortality outcomes following head trauma sustained in a combat setting between ketamine recipients and non-recipients.

Methods

This is a secondary analysis of previously published data in the Department of Defense Trauma Registry from January 2007 to August 2016. We isolated patients with an abbreviated injury scale of 3 or greater for the head body region. We compared mortality between prehospital ketamine recipients and non-recipients.

Results

Our initial search yielded 28,222 patients, of which 4,183 met the inclusion criteria: 209 were ketamine-recipients and 3,974 were non-recipients. The ketamine group had a higher percentage injured by explosives (59.81% vs. 53.57%, p<0.001) and gunshot wounds (28.71% vs. 22.07%, p<0.001) and were more frequently located in Afghanistan (100% vs. 68.0%, p<0.001). The ketamine group had higher rates of tourniquet application (24.4% vs. 8.5%, p<0.001) and had lower survival proportion (75.1% alive vs. 83.0%, p=0.003). All differences were significant. On univariable analysis, the ketamine group had worse odds of survival with (OR: 0.62; 95%CI: 0.45-0.86). When controlling for the presence of an airway intervention and mechanism of injury, the finding was non-significant (OR: 1.09; 95% CI: 0.76-1.55).

Conclusions

In our prehospital combat study, after controlling for confounders, we found no association between administration of prehospital ketamine and worse survival outcomes for casualties with head injuries. However, despite the lack of difference in overall survival noted, those who received ketamine and died had a higher risk ratio for time to death.

## Introduction

Pain management in the prehospital setting plays a key role in patient prognosis, including physiological and psychological long-term complications. The military combat prehospital setting provides additional challenges for analgesic administration due to the austere environment, prolonged transport times, and often multiple casualties [1]. The U.S. military relies on the Tactical Combat Casualty Care (TCCC) guidelines to propagate best practices in battlefield medical care [2]. TCCC guidelines recommend ketamine for analgesia for “casualties who have moderate-to-severe pain, but who are in hemorrhagic shock or respiratory distress or are at significant risk for developing either condition” [3]. Ketamine is unique in its ability to provide rapid pain relief with a wide therapeutic window while minimally affecting central respiratory drive [4-6]. Uniquely, ketamine preserves airway reflexes and does not compromise airway integrity at high doses [7]. However, ketamine can cause increases in blood pressure and potentially change intracranial hemodynamics [8].

TCCC recommends against ketamine usage in patients with severe traumatic brain injuries (TBI) due to concerns with ketamine inducing increased intercranial pressure (ICP) [9]. However, there are few published studies on prehospital ketamine use and TBI outcomes to support or refute this caution. In fact, recent studies suggest that these changes may have no clinical effect [10]. The need to determine the validity of this concern is compounded by the difficulty to assess head trauma in the prehospital setting [11], the limited options for pharmaceutical analgesics in the austere environment of the battlefield [1], and the lack of adherence to TCCC analgesia guidelines in the prehospital setting [12]. This lack of adherence complicates efforts to validate the absence of association between increased ICP and ketamine use, as seen in the available data.

Thus, we propose to add data of prehospital ketamine use and TBI outcomes specifically relevant to the battlefield by using information gathered from combat casualty care.

Goal of this study

Our aim is to describe mortality after initial hospitalization of combat patients with TBI who received ketamine in the prehospital setting compared to those who did not.

## Materials and methods

Data acquisition

**Figure 1:**
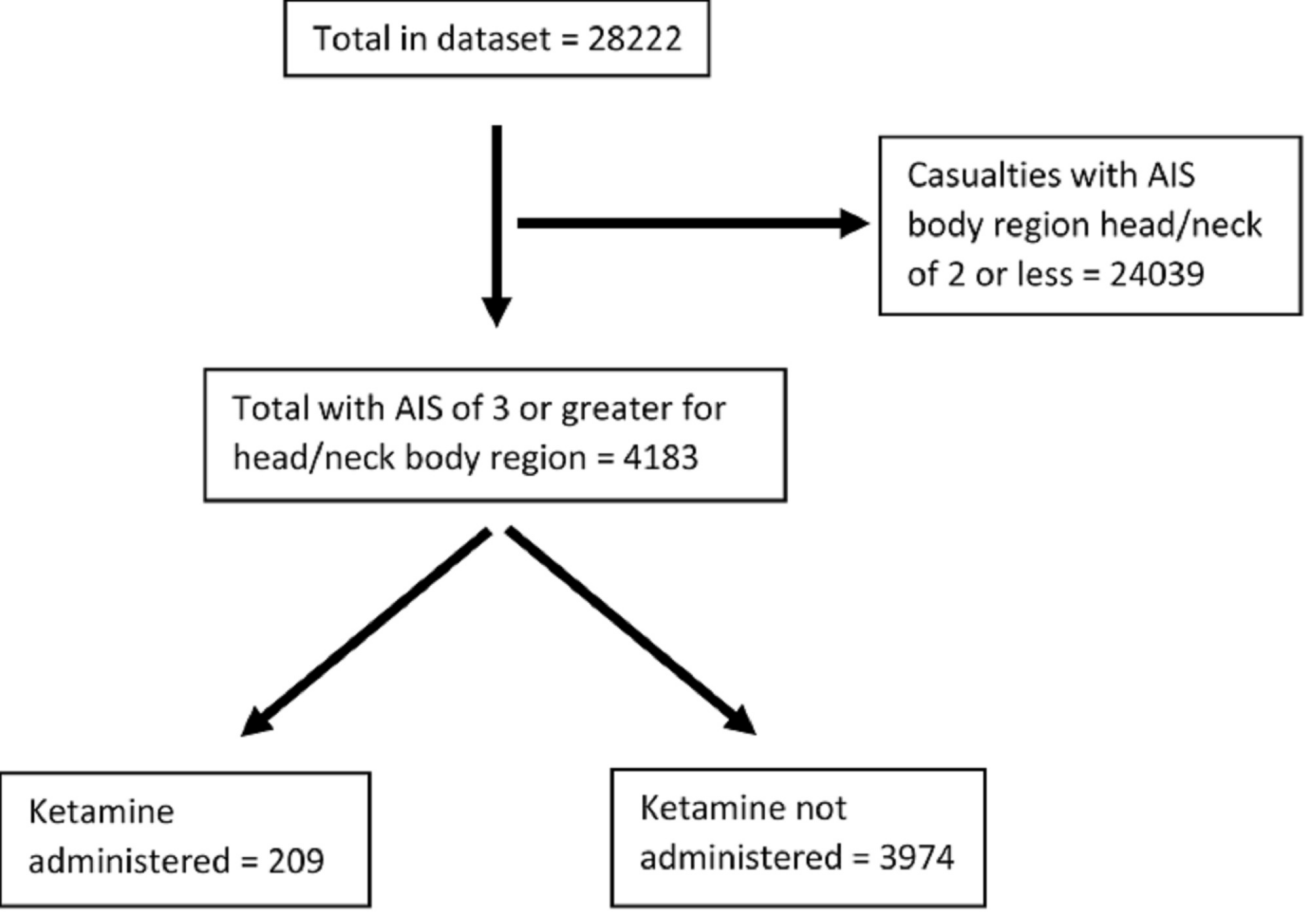
Flow chart demonstrating how patients were chosen Y-axis: alive (1) versus dead (0) AIS, abbreviated injury scale

We conducted a retrospective review of prospectively collected data in the Department of Defense Trauma Registry (DoDTR) between January 2007 and August 2016. This is a secondary analysis of a previously described dataset in which we isolated patients who sustained head trauma based on an abbreviated injury scale (AIS) of 3 or greater for the head body region (Figure 1) [13]. The U.S. Army Institute of Surgical Research regulatory office reviewed protocol H-16-005 and determined the protocol was exempt from institutional review board oversight. We obtained only de-identified data.

Department of Defense Trauma Registry description

The DoDTR, formerly known as the Joint Theater Trauma Registry (JTTR), is the data repository for the Department of Defense trauma-related injuries. The DoDTR includes documentation regarding demographics, injury-producing incidents, diagnoses, treatments, and outcomes of injuries sustained by U.S. military and U.S. civilian personnel in wartime and peacetime from the point of injury to final disposition. Short-term outcome data are available for non-U.S. casualties. The DoDTR comprises all patients admitted to a role 3 (fixed-facility) or forward surgical team (FST) with an injury diagnosis using the International Classification of Disease 9th Edition (ICD-9) between 800 and 959.9, near-drowning/drowning with associated injury (ICD-9 994.1) or inhalational injury (ICD-9 987.9) and trauma occurring within 72 hours from injury. The registry defines the prehospital setting as any location prior to reaching an FST or a combat support hospital to include role 1 (point of injury, casualty collection point, battalion aid station) and role 2 (temporary limited-capability forward-positioned hospital inside combat zone without surgical support, excludes the joint definition role 2e) [6,13-15].

Analysis

We performed all statistical analysis using Microsoft Excel (version 10, Microsoft Corp., Redmond, Washington) and JMP Statistical Discovery from SAS (version 13, SAS Institute Inc., Cary, NC). We evaluated study variables including interventions and outcomes between males and females. We compared continuous variables using Student’s t-test or Wilcoxon’s rank sum test and reported findings as mean with standard deviations and as median with interquartile range, respectively. We compared categorical data using the chi-square test and reported findings as counts and percentages. Finally, we compared study variables using univariable and multivariable logistic regression models (MVLRs) designed to control for potential confounders. Our model was based on factors that have been previously linked to poor outcomes (e.g. airway interventions) and the need for airway interventions as ketamine is frequently used as a sedative agent during rapid sequence intubation [16,17]. Significant variables were reported as odds ratios with 95% confidence intervals (OR, 95% CI). We placed all airway interventions into one category (e.g. nasopharyngeal airway, oropharyngeal airway, intubation, cricothyrotomy). Statistical significance was set at p < 0.05. Extremity amputations were included if they were proximal to the digits. We used the same models described in the MVLR and performed additional testing including the Kaplan-Meier curve with log ranks test for time-to-event analyses and a Cox proportional test to determine the hazards ratio with the covariables described in the Results section.

## Results

Our initial search codes identified 28,222 patients in the DoDTR, which has been previously described [13]. Of the 28,222 patients, 4,183 had an AIS score of 3 or greater for the head body region, of whom 209 received prehospital ketamine and 3,974 did not. Casualties receiving ketamine had a statistically significant, but clinically non-significant, and lower median age (24 vs. 25 years). Most of the casualties from both cohorts were host nation forces (ketamine 41.1% vs. control 33.1%, p<0.001), were in Afghanistan (ketamine 100% vs. control 68.0%, p<0.001), and injured by explosive (ketamine 59.8% vs. control 53.5%, p<0.001). Composite injury severity scores (ISSs) were similar (22 vs. 22) as were nearly all AIS by body region except for significantly higher median AIS for the extremities (1 vs. 0, p<0.001). Ketamine recipients had significantly lower survival to hospital discharge (75.1% vs. 83.0%, p = 0.003; Table 1). Airway interventions occurred in a higher proportion of ketamine recipients (46.4% vs. 12.8%, p<0.001). A higher proportion of ketamine recipients had an amputation (13.8% vs. 4.9%, p<0.001) as was tourniquet placement (24.4% vs. 8.5%, p<0.001).

**Table 1:** Comparison of casualties with head trauma ketamine recipients versus ketamine non-recipients MVC, motor vehicle collision; AIS, abbreviated injury scale

		Ketamine Cohort (n=209)	Control Cohort (n=3,974)	p-Value
Demographics	Median age, years	24 (21-30)	25 (21-31)	0.017
	Male	99.5% (208)	96.8% (3,845)	0.021
Patient category	U.S. military	20.1% (42)	26.9% (1,072)	<0.001
	Coalition	9.1% (19)	4.6% (184)	
	Host nation forces	41.1% (86)	33.1% (1,318)	
	Humanitarian	27.2% (57)	29.8% (1,187)	
	Other	2.4% (5)	5.3% (213)	
Country	Afghanistan	100% (209)	68.0% (2,703)	<0.001
	Iraq	0% (0)	31.9% (1,271)	
Mechanism of injury	Explosive	59.8% (125)	53.5% (2,129)	<0.001
	Gunshot wound	28.7% (60)	22.1% (877)	
	MVC	8.1% (17)	15.1% (600)	
	Other	3.3% (7)	9.2% (368)	
Injury score	Composite	22 (16-29)	22 (14-29)	0.169
	AIS (face)	1 (0-2)	1 (0-2)	0.247
	AIS (thorax)	0 (0-2)	0 (0-2)	0.237
	AIS (abdomen)	0 (0-1)	0 (0-0)	0.061
	AIS (extremities)	1 (0-3)	0 (0-2)	<0.001
	AIS (skin/superficial)	1 (0-1)	1 (0-1)	0.087
Outcome	Discharged alive	75.1% (157)	83.0% (3,296)	0.003

On univariable analysis, the odds of survival were lower in ketamine recipients (OR: 0.61; 95% CI: 0.44-0.85). When adjusting for any airway intervention, mechanism of injury, and composite ISS to the model, the finding was non-significant (OR: 1.07; 95% CI: 0.75-1.53; Table 2).

**Table 2:** Logistic regression model permutations with model component odds for survival to discharge All data presented as the odds ratio and 95% confidence intervals. *Presented as odds ratio. GSW, gunshot wound; MVC, motor vehicle collision

Ketamine 0.61 (0.44-0.85)
Ketamine 1.06 (0.74-1.50)
Any airway 0.23 (0.19-0.28)
Ketamine 1.09 (0.76-1.55)
Any airway 0.25 (0.20-0.31)
Explosive/GSW 2.28 (1.89-2.76)
Explosive/MVC 1.17 (0.91-1.50)
Explosive/other 0.46 (0.30-0.71)
Ketamine 1.07 (0.75-1.53)
Any airway 0.28 (0.22-0.34)
Explosive/GSW 2.64 (2.17-3.21)
Explosive/MVC 1.33 (1.03-1.73)
Explosive/other 0.55 (0.36-0.85)
Injury severity score* 0.95 (0.96-1.04)

**Figure 2:**
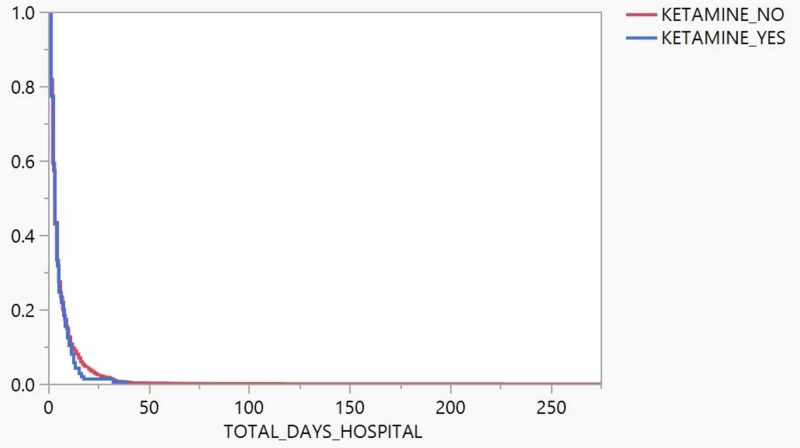
Kaplan-Meier time-to-event analysis between groups (p=0.207)

On the Kaplan-Meier curve using the same developed model, we found no difference in the time-to-event analysis for deaths (Figure 2, p=0.207). In a proportional hazards model for time to death without adjustment, the risk ratio for ketamine recipients versus non-recipients was 1.09 (95% CI: 0.93-1.28). With adjustment for airway intervention and mechanism of injury, the risk ratio for ketamine recipients versus non-recipients was 1.18 (95% CI: 1.00-1.39). When adjusting the model to also include ISS, the risk ratio for ketamine recipients versus non-recipients was 1.20 (95% CI: 1.02-1.42).

## Discussion

Our study found no statistically significant difference with regard to mortality outcomes of ketamine recipients and non-recipients when controlling for confounders. The ketamine recipient group experienced a higher severity of injuries compared to the control group. It also appeared that ketamine was more frequently administered to this population in Afghanistan, which is likely due to differences in supply chain and adoption of theater policy changes [18].

Early studies of ketamine use reported a possible rise in ICP [19,20] and led to recommendations of brain injury as a contraindication to ketamine use. Thus, the use of ketamine after combat injuries, specifically explosive injuries that may have a concomitant brain injury, has been controversial. However, more recent pediatric studies have reported wide margins of safety for ketamine administration based on pediatric populations [6]. They have also found no mortality difference between ketamine and other pharmaceuticals for anesthesia induction [21-23]. In addition, ketamine administration was also not found to increase ICP compared to opioids or to adversely affect mortality in several meta-analyses [24,25]. These studies do not include information on prehospital administration of ketamine and/or combat use and subsequent mortality outcomes. Our study is specifically important to the military as it includes a preponderance of explosive-based injuries, something rarely seen in the civilian setting. Our study provides novel information on mortality outcomes for adult populations given analgesic doses of ketamine in the prehospital setting, resulting in no differences, albeit it is only one drug in a long course of care. While we could not measure ICP in our cases, we could measure several clinical outcomes immediately after injury survival to hospital discharge.

Our dataset allowed our study to be one of the largest studies of prehospital ketamine for TBI patients. Our results support the current literature from the civilian population and are a preliminary step toward refuting the warning advising avoidance of ketamine in the setting of TBI within the TCCC guidelines. They also indicate a need for prospective studies.

In the future, the field would benefit from additional patient-centered outcomes beyond mortality such as post-traumatic stress disorder (PTSD), chronic headaches, disability, and others similar to previous studies evaluating early use of morphine after combat injury [26]. Moreover, findings specific to those with mild TBI would further enhance the ability to understand the optimum patient-population for ketamine use.

Limitations

This study is limited due to the use of the retrospective design of the study. Our dataset is primarily male and military-affiliated, which may limit the applicability to other populations. The database did not include specific ketamine indications; thus, we are unable to separate out those receiving ketamine for rapid-sequence intubation versus analgesia or anxiety. Moreover, we do not know when in their course of care the drug was administered relative to other interventions. Our study focused on mortality differences; therefore, it remains unclear whether the same findings would apply to other end-points, such as changes in ICP or more long-term outcomes such as neurologic function at discharge, functional outcomes, chronic headaches, or PTSD [27,28]. Our data do not include shock index or other such confounders. The data are also limited in its lack of additional outcomes, such as quality of life or clinical course. Lastly, patients are included in the registry with missing data (including unknown omissions of drug administration), which may have effects on data quality. Previous studies demonstrate poor documentation in the prehospital combat setting, with repeated calls for higher quality data capture from the prehospital setting [29,30]. Our findings should be further validated across other data capture systems and perhaps other combat settings.

## Conclusions

In our prehospital combat study, after controlling for confounders, we found no association between administration of prehospital ketamine and worse survival outcomes for casualties with head injuries. However, despite the lack of difference in overall survival noted, those who received ketamine and died had a higher risk ratio for time to death. Our study is one of the largest studies of prehospital ketamine for TBI patients.

## References

[1] Benov A, Salas MM, Nakar H, Antebi B, Tarif B, Yitzhak A, Glassberg E (2017). Battlefield pain management: a view of 17 years in Israel Defense Forces. J Trauma Acute Care Surg.

[2] Butler FK (2017). Two decades of saving lives on the battlefield: Tactical Combat Casualty Care turns 20. Mil Med.

[3] Butler FK, Kotwal RS, Buckenmaier III CC (2014). A triple-option analgesia plan for Tactical Combat Casualty Care: TCCC Guidelines Change 13-04. J Spec Oper Med.

[4] Wedmore IS, Butler FK (2017). Battlefield analgesia in Tactical Combat Casualty Care. Wilderness Environ Med.

[5] Li L, Vlisides PE (2016). Ketamine: 50 years of modulating the ind. Front Hum Neurosci.

[6] Green SM, Rothrock SG, Lynch EL (1998). Intramuscular ketamine for pediatric sedation in the emergency department: safety profile in 1,022 cases. Ann Emerg Med.

[7] Green SM, Roback MG, Kennedy RM, Krauss B (2011). Clinical practice guideline for emergency department ketamine dissociative sedation: 2011 update. Ann Emerg Med.

[8] Zeiler FA, Teitelbaum J, West M, Gillman LM (2014). The ketamine effect on ICP in traumatic brain injury. Neurocrit Care.

[9] Chang LC, Raty SR, Ortiz J, Bailard NS, Mathew SJ (2013). The emerging use of ketamine for anesthesia and sedation in traumatic brain injuries. CNS Neurosci Ther.

[10] Farrell D, Bendo AA (2018). Perioperative management of severe traumatic brain injury: what is new?. Curr Anesthesiol Rep.

[11] Committee on the Assessment of the Readjustment Needs of Military Personnel, Veterans Veterans, and Their Families; Board on the Health of Select Populations; Institute of Medicine (2013). Returning Home from Iraq and Afghanistan: Assessment of Readjustment Needs of Veterans, Service Members, and Their Families. https://www.ncbi.nlm.nih.gov/books/NBK206875/..

[12] Schauer SG, Robinson JB, Mabry RL, Howard JT (2015). Battlefield analgesia: TCCC guidelines are not being followed. J Spec Oper Med.

[13] Schauer SG, Naylor JF, Oliver JJ, Maddry JK, April MD (2019). An analysis of casualties presenting to military emergency departments in Iraq and Afghanistan. Am J Emerg Med.

[14] Schauer SG, April MD, Hill GJ, Naylor JF, Borgman MA, Lorenzo RAD (2018). Prehospital interventions performed on pediatric trauma patients in Iraq and Afghanistan. Prehosp Emerg Care.

[15] Schauer SG, Hill GJ, Naylor JF, April MD, Borgman M, Bebarta VS (2018). Emergency department resuscitation of pediatric trauma patients in Iraq and Afghanistan. Am J Emerg Med.

[16] Blackburn MB, April MMD, Brown CDJ, DeLorenzo RA, Ryan KL, Blackburn AN, Schauer SG (2018). Prehospital airway procedures performed in trauma patients by ground forces in Afghanistan. J Trauma Acute Care Surg.

[17] Schauer SG, Naylor JF, Maddry JK (2018). Prehospital airway management in Iraq and Afghanistan: a descriptive analysis. South Med J.

[18] Schauer SG, Naylor JF, Maddry JK, Hinojosa-Laborde C, April MD (2019). Trends in prehospital analgesia administration by US forces from 2007 through 2016. Prehosp Emerg Care.

[19] Green SM, Andolfatto G, Krauss BS (2015). Ketamine and intracranial pressure: no contraindication except hydrocephalus. Ann Emerg Med.

[20] Zeiler FA, Teitelbaum J, West M, Gillman LM (2014). The ketamine effect on ICP in traumatic brain injury. Neurocrit Care.

[21] Upchurch CP, Grijalva CG, Russ S (2017). Comparison of etomidate and ketamine for induction during rapid sequence intubation of adult trauma patients. Ann Emerg Med.

[22] Jabre P, Combes X, Lapostolle F (2009). Etomidate versus ketamine for rapid sequence intubation in acutely ill patients: a multicentre randomised controlled trial. Lancet.

[23] Grathwohl KW, Black IH, Spinella PC (2008). Total intravenous anesthesia including ketamine versus volatile gas anesthesia for combat-related operative traumatic brain injury. Anesthesiology.

[24] Wang X, Ding X, Tong Y, Zong J, Zhao X, Ren H, Li Q (2014). Ketamine does not increase intracranial pressure compared with opioids: meta-analysis of randomized controlled trials. J Anesth.

[25] Cohen L, Athaide V, Wickham ME, Doyle-Waters MM, Rose NG, Hohl CM (2015). The effect of ketamine on intracranial and cerebral perfusion pressure and health outcomes: a systematic review. Ann Emerg Med.

[26] Holbrook TL, Galarneau MR, Dye JL, Quinn K, Dougherty AL (2010). Morphine use after combat injury in Iraq and post-traumatic stress disorder. N Eng J Med.

[27] McGhee LL, Maani CV, Garza TH, Gaylord KM, Black IH (2008). The correlation between ketamine and posttraumatic stress disorder in burned service members. J Trauma.

[28] McGhee LL, Maani CV, Garza TH, Slater TM, Petz LN, Fowler M (2014). The intraoperative administration of ketamine to burned U.S. service members does not increase the incidence of post-traumatic stress disorder. Mil Med.

[29] Schauer SG, April MD, Naylor JF (2017). A descriptive analysis of data from the Department of Defense Joint Trauma System Prehospital Trauma Registry. US Army Med Dep J.

[30] Robinson JB, Smith MP, Gross KR, Sauer SW, Geracci JJ, Day CD, Kotwal RS (2016). Battlefield documentation of Tactical Combat Casualty Care in Afghanistan. US Army Med Dep J.

